# Systematic Postoperative Assessment of a Minimally-Invasive Sheep Model for the Treatment of Osteochondral Defects

**DOI:** 10.3390/life10120332

**Published:** 2020-12-07

**Authors:** Long Xin, Joerg Mika, Victoria Horbert, Sabine Bischoff, Harald Schubert, Juliane Borowski, Stefan Maenz, René Huber, Andre Sachse, Bernhard Illerhaus, Raimund W. Kinne

**Affiliations:** 1 Experimental Rheumatology Unit, Department of Orthopedics, Jena University Hospital, Waldkliniken Eisenberg GmbH, 07607 Eisenberg, Germany; kjb@zjtongde.com (L.X.); joergmika@yahoo.com (J.M.); victoria.horbert@med.uni-jena.de (V.H.); juliane.borowski@gmx.de (J.B.); 2 Department of Orthopedics, Tongde Hospital of Zhejiang Province, Hangzhou 310012, China; 3 Institute of Laboratory Animal Sciences and Welfare, Jena University Hospital, 07607 Jena, Germany; sabine.bischoff@med.uni-jena.de (S.B.); harald.schubert@outlook.de (H.S.); 4 Chair of Materials Science, Otto Schott Institute of Materials Research, Friedrich-Schiller-University, 07743 Jena, Germany; stefanmaenz@aol.com; 5 Institute of Clinical Chemistry, Hannover Medical School, 30625 Hannover, Germany; huber.rene@mh-hannover.de; 6 Department of Orthopedics, Jena University Hospital, Waldkliniken Eisenberg GmbH, 07607 Eisenberg, Germany; a.sachse@waldkliniken-eisenberg.de; 7 Bundesanstalt für Materialforschung und -Prüfung (BAM), 12205 Berlin, Germany; illerhaus@berlin.de

**Keywords:** osteochondral stifle joint defect, sheep animal model, minimally-invasive parapatellar approach

## Abstract

To assess the clinical course of a sheep stifle joint model for osteochondral (OC) defects, medial femoral condyles (MFC) were exposed without patella luxation using medial parapatellar skin (3–4 cm) and deep incisions (2–3 cm). Two defects (7 mm diameter; 10 mm depth; OC punch) were left empty or refilled with osteochondral autologous transplantation cylinders (OATS) and explanted after six weeks. Incision-to-suture time, anesthesia time, and postoperative wound or impairment scores were compared to those in sham-operated animals. Implant performance was assessed by X-ray, micro-computed tomography, histology, and immunohistology (collagens 1, 2; aggrecan). There were no surgery-related infections or patellar luxations. Operation, anesthesia, and time to complete stand were short (0.5, 1.4, and 1.5 h, respectively). The wound trauma score was low (0.4 of maximally 4; day 7). Empty-defect and OATS animals reached an impairment score of 0 significantly later than sham animals (7.4 and 4.0 days, respectively, versus 1.5 days). Empty defects showed incomplete healing and dedifferentiation/heterotopic differentiation; OATS-filled defects displayed advanced bone healing with remaining cartilage gaps and orthotopic expression of bone and cartilage markers. Minimally-invasive, medial parapatellar surgery of OC defects on the sheep MFC allows rapid and low-trauma recovery and appears well-suited for implant testing.

## 1. Introduction

Osteochondral (OC) lesions are found in 61% of patients with joint pain [1]. If untreated, these lesions can lead to the long-term development of full clinical osteoarthritis (OA), and, therefore, timely treatment is required [1,2,3,4,5,6,7]. Several small and large animal models have been described for OC defects [8,9], but large animals represent more closely the human situation and are, therefore, recommended by the regulatory bodies [10]. Particularly the ovine stifle joint is frequently used to study OC defects due to anatomical, biochemical, and biomechanical similarities to the human knee joint [9,11,12].

The medial femoral condyle (MFC; [13,14]) and the trochlear groove [14,15] are most commonly studied in the ovine stifle joint. Traditional surgical approaches include large arthrotomy incisions and luxation or subluxation of the patella. These procedures, however, may damage the medial patellar retinaculum or parts of the quadriceps muscle, with subsequent permanent muscle damage or postoperative patella dislocation [13,16]. In addition, sometimes two-step defect models are used for tissue harvest, defect generation, and/or tissue engineering, possibly increasing pain and impairing joint function [17,18,19].

More recently, less invasive arthrotomy techniques without patella luxation have been developed to limit tissue damage and favor rapid rehabilitation of the animals [17,18,19,20]. In particular, a minimally-invasive, low-morbidity surgical access to the trochlea/MFC of the ovine stifle joint was described [20], however, with limited details on intra-operative and postoperative parameters.

Thus, this study was undertaken to systematically assess the stifle joint sheep animal model for OC defect therapy, including surgical and behavioral details and a sham group to differentiate the effects of surgical procedure and defect generation. Critical-size empty and osteochondral autologous transplantation cylinder (OATS-) filled defects were compared as a proof of concept for implant healing. In the present study, empty defects were not only used as critical-size controls but also as follow-up locations for donor-site morbidity following OATS [21,22,23,24,25].

## 2. Materials and Methods

### 2.1. Animals

A total of 44 healthy, skeletally mature sheep were included (*n* = 44 Merino animals; age 3.26 ± 1.10 years; body weight 65.0 ± 9.2 kg; means ± standard deviations).

Surgical and behavioral details of the new model were assessed until postoperative day 14 in 32 animals operated in the right stifle joint (2 empty defects; 2 osteochondral autologous transplantations, OATS; *n* = 16 animals each) as negative or positive controls for unpublished and unrelated previous studies of experimental OC repair (permission number 02-029/14; governmental commission for animal protection, Free State of Thuringia, Germany). In addition, X-rays were obtained from 2 selected animals (one each with 2 empty defects and 2 OATS transplants) 6 weeks after surgery. The animals of these 2 groups were later sacrificed (after 6 and 12 months; data not shown) by i.v. injection of overdosed barbiturate (Pentobarbital™, Essex Pharma GmbH, Munich, Germany).

Four additional animals were sacrificed after 6 weeks with permission from an experiment to train the surgical technique (permission number 02-135/13) to obtain experimental data for micro-computed tomography (micro-CT), gross morphological observations, and histological evaluation. These animals received either 1 empty defect and 1 OATS or, alternatively, 2 OATS in the right stifle joint (*n* = 2 animals each) and were also sacrificed by i.v. injection of overdosed barbiturate (Pentobarbital™, Essex Pharma GmbH, Munich, Germany).

Finally, an additional sham group with 8 animals (only exposure of the MFC, but without the creation of OC defects or implantation) was operated on to comparatively assess intra-operative and postoperative surgical/behavioral parameters (until postoperative day 14). In line with the respective permission, this group was not euthanized (permission number 02-029/14).

### 2.2. Anesthesia and Surgical Technique

**Figure 1:**
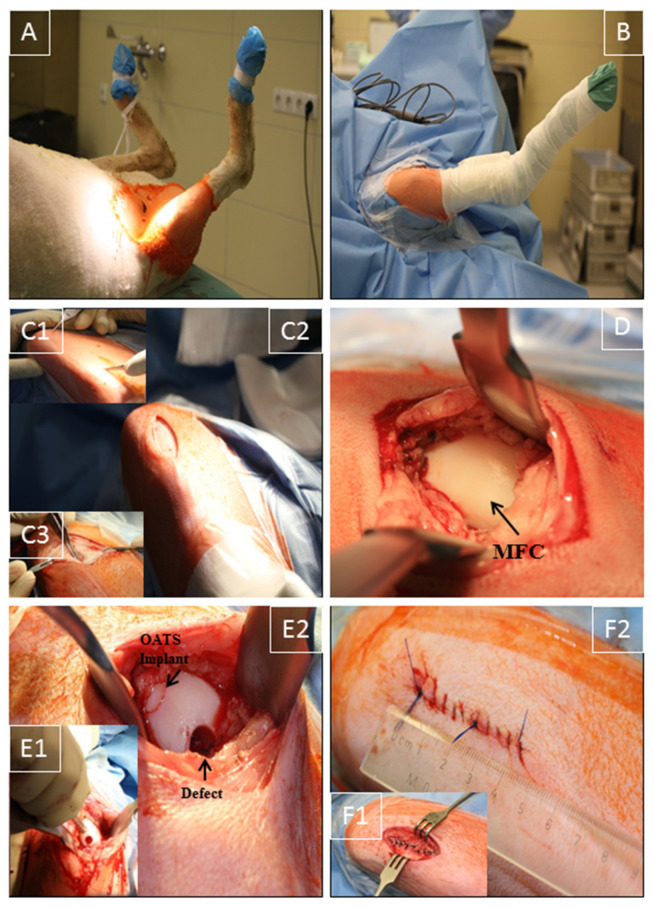
Surgical technique. (**A**) Supine positioning of the sheep; (**B**) operation situs following disinfection and sterile covering of claw and lower limb by a surgical drape/bandage; (**C1**,**2**) medial, 3–4 cm long, parapatellar skin incision from the distal patella pole to a level approximately 2 cm proximal of the tuberositas tibiae; (**C3**) smaller medial incision (2–3 cm) through the subcutaneous tissue and joint capsule; (**D**) exposure of the medial femoral condyle (MFC) without luxation of the patella; (**E1**,**2**) maximal flexion of the stifle joint to expose the distal MFC weight-bearing region; creation of 2 defects (7 mm diameter and 10 mm depth) using a standardized OC punch (**E1**); empty defect (empty control) or defect refilled with the original OATS cylinder (**E2**); (**F1**,**2**) layer-wise suturing of synovial capsule (interrupted sutures), subcutaneous tissue, and skin (continuous sutures; length approximately 4 cm).

The right stifle joint of the animals was shaved under sedation, and anesthesia was performed as previously published [26,27]. The sheep were placed in the supine position (Figure 1A), and the claw and lower limb were covered by a surgical drape and a bandage. Two U-shaped surgical drapes were used to create an operating field of approximately 15 cm around the stifle joint (Figure 1B).

Using the anatomical landmarks distal patella pole, tuberositas tibiae, and fossa intercondylaris, a medial, 3–4 cm long, parapatellar skin incision was placed from the distal patella pole to a level approximately 2 cm proximal of the tuberositas tibiae (Figure 1C1,C2). Without patella luxation, a smaller, muscle-sparing, medial incision (2–3 cm) through subcutaneous tissue and joint capsule at the border of the patellar tendon was applied to expose the MFC, using 2 Hohmann retractors and limited resection of the infrapatellar fat pad. Great care was taken to avoid damage to cartilage, quadriceps muscle, or medial patellar retinaculum (Figure 1C3,D), in direct analogy to the human knee joint procedure [28,29]. Diathermy was used to control bleeding, and the stifle joint was maximally flexed to expose the distal weight-bearing region of the MFC (Figure 1D). Two defects (7 mm diameter; 10 mm depth) were created using standardized OC punches perpendicular to the tangent plane of the MFC (Single-use OATS set, 6 mm, AR-1981-06S, Arthrex, Munich, Germany; Figure 1E1), and either left empty (empty control) or refilled with the original OATS cylinder (OATS; Figure 1E2). For this purpose, the screw-in core extruder was removed from the 6 mm diameter donor harvester of the set, and the sharp front edge of the harvester was first perpendicularly anchored in the cartilage of the defect site by gentle tapping with a mallet. The donor harvester was subsequently impacted to a graduated depth of 10 mm, and the OC cylinder was then disengaged from the subchondral bone by 2 rapid 90° clockwise and counter-clockwise turns of the harvester. For the OATS-filled defects, the OC cylinder was expelled from the harvester by advancing the screw-in core extruder and replaced loose-fit into the donor defect. This procedure was chosen to avoid the generation of additional defects in the operated stifle joint. Reliable placement of the OATS cylinder was checked by the 10-time articulation of the joint. For the sham group (*n* = 8), exposure of the MFC was performed as above, but without the creation of OC defects or implantation; to adjust the operating time, a waiting time of 10 min was introduced after full exposure of the MFC.

Synovial capsule (interrupted sutures; 2-0 Vicryl, Ethicon, Norderstedt, Germany), subcutaneous tissue (2-0 Vicryl), and skin (Prolene, Ethicon; both continuous sutures) were then closed in layers (resulting in skin suture approximately 4 cm; Figure 1F1,2). Surgery was performed in <30 min with minimal postoperative discomfort (Figure 1). After placing an adhesive bandage, the right leg was immobilized with a fiberglass cast (1 week).

After surgery, animals were housed in separate pens (1–2 weeks) and then returned to long-care paddocks (4 weeks), with postoperative intramuscular medication as published [26,27].

### 2.3. Assessment of Operation Duration and Postoperative Recovery

Intra-operative incision-to-suture/anesthesia time, postoperative wound and impairment scores, and time to complete stand without lying down again were documented. The wound score was adapted from a 5-level macroscopic system developed for cattle [30], with 0 = no signs of wound secretion, swelling or infection; 1 = signs of wound secretion; 2 = signs of wound swelling/redness; 3 = signs of swelling/redness and wound secretion; 4 = signs of wound secretion, swelling/redness, and infection. Skin sutures were removed after 10 days.

A validated 5-level impairment score was used [31,32], with 0 = animal stands and walks normally; 1 = stands normally, slight lameness while walking; 2 = stands normally, severe lameness while walking; 3 = abnormal posture when standing, severe lameness when walking; 4 = does not bear weight on the surgical limb at rest or when walking.

### 2.4. Radiographic Analysis

Implant performance was judged in 2 animals by in vivo X-ray after 6 weeks (Optimus 50; Philips GmbH; Hamburg, Germany), comparing the contralateral, non-operated left stifle joint (normal control), empty MFC defects (empty defect), and the integrated OATS in the defect.

### 2.5. High-Resolution Micro-CT

Micro-CT analysis was performed as previously published [27]. A total of 8 frozen stifle joints from *n* = 4 sheep were analyzed under dry ice within 6 h, resulting in a voxel size of 66.6 µm.

### 2.6. Gross Morphological Observation and Histological Evaluation

After excision of osteochondral blocks (size 4 × 2 × 2.5 cm; length × width × depth) with an oscillating bone saw (6 weeks post-surgery), the MFC surface of 8 defects in *n* = 4 sheep were macroscopically evaluated, and the samples were then stored at −20 °C.

Following decalcification of the samples, paraffin sections of 7 μm thickness were then cut and stained with hematoxylin and eosin (HE), trichrome stain according to Masson-Goldner, Safranin-O, or Toluidine blue (the latter 2 stainings were performed to assess the proteoglycan content).

For aggrecan immunohistology, slices were treated with chondroitinase ABC (Sigma-Aldrich, St. Louis, MO, USA; 0.25 U/mL; 37 °C; 90 min), blocked with H_2_O_2_ and 10% goat serum/tris-buffered saline (TBS), and incubated overnight at 4 °C with the antibody GTX75039 (GenTex, Irvine, CA, USA; 1:150).

For collagen I and II immunohistology, epitopes were demasked with Proteinase K (code S3004; Dako, Hamburg, Germany; 1:50; room temperature (RT); 15 min), blocked first with 0.5% H_2_O_2_ (in methanol; 10 min), and then with 25% normal bovine serum (BSA)/tris-buffered saline (30 min; RT), and incubated overnight at 4 °C with primary antibodies to bovine collagen I (polyclonal rabbit immunoglobulin (Ig) G; Acris, Herford, Germany; 2 µL/mL) or collagen II (polyclonal rabbit IgG; Acris; 10 µg/mL).

This was followed by incubation for 1 h at room temperature (RT) with a secondary antibody (anti-mouse or anti-rabbit) coupled to horseradish peroxidase (HRP; for collagen 1 and 2 antibodies) or alkaline phosphatase (ALP; for aggrecan) and visualization of HRP with diaminobenzidine (DAB) and ALP with Fast Red (both Sigma Aldrich). Sections were then counterstained with hematoxylin and mounted with Aquatex (Merck, Darmstadt, Germany).

For collagen I/collagen II double-immunohistology, the above-mentioned staining for collagen I was followed by detection with a secondary HRP goat anti-rabbit IgG antibody for 1 h at RT and visualization with DAB (Sigma-Aldrich). Thereafter, staining for collagen II, incubation with a secondary ALP goat anti-mouse IgG antibody (1 h; RT), and visualization by Fast Red was performed (Sigma-Aldrich) without nuclear counterstaining.

In single or double-immunohistology stainings, isotype-matched control immunoglobulins consistently yielded negative results.

### 2.7. Statistical Analysis

Data were expressed as means ± standard deviations. Data were first analyzed using the non-parametric multi-group Kruskal–Wallis test (using *p* ≤ 0.01 as significance level to address the problem of multiple testing by reducing the number of statistical comparisons); significant differences between individual groups were then tested using the Mann–Whitney U test (*p* ≤ 0.05; SPSS version 22; SPSS Inc., Chicago, IL, USA).

## 3. Results

### 3.1. Operation Time and Postoperative Recovery

**Figure 2:**
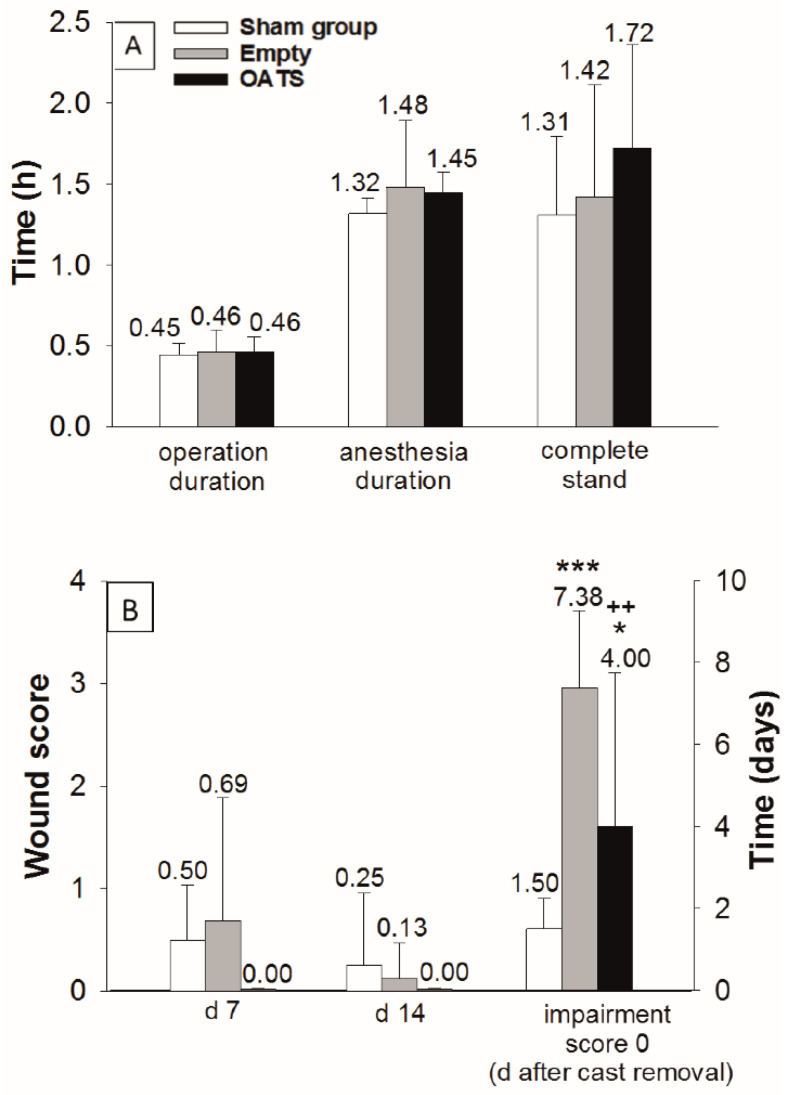
Assessment of surgical/clinical parameters in sham (*n* = 8), empty defect, and OATS groups (*n* = 16 each). Operation/anesthesia duration and time to complete stand (**A**), as well as postoperative wound score (days 7 and 14), and time to return to an impairment score of 0 after cast removal (**B**); data represent the means ± standard deviations; *** *p* ≤ 0.001; * *p* ≤ 0.05 versus sham group; ++ *p* < 0.01 versus empty defect group.

In empty defect and OATS groups (*n* = 16 animals each), the duration of operation (both 28 min; 0.46 h) and total anesthesia was limited (89 and 87 min; 1.48 h and 1.45 h, respectively; Figure 2A). Due to the waiting time after exposure of the MFC (10 min), the sham group (*n* = 8 animals) showed an almost identical operation time (27 min; 0.45 h) and a slightly reduced anesthesia duration (79 min; 1.32 h; Figure 2A).

The operation was minimally-invasive, as shown by: (a) Very short postoperative recovery times to complete stand (sham: 1.31 h; empty defect: 1.42 h; and OATS: 1.72 h; Figure 2A); (b) limited postoperative wound scores (day 7: 0.50, 0.69, and 0.00; day 14: 0.25, 0.13, and 0.00; Figure 2B); and (c) limited periods to reach a postoperative impairment score of 0 after cast removal (1.50, 7.38, and 4.00 days). The sham group significantly differed from the empty defect and OATS groups only in the time to reach an impairment score of 0 (*p* ≤ 0.001 versus empty defect; *p* ≤ 0.05 versus OATS). Interestingly, this parameter also significantly differed between empty defect and OATS groups (*p* ≤ 0.01; Figure 2B).

No surgery-related infections, patellar luxations, or other complications were observed. All animals returned to normal behavior, gait, and food and water consumption within two weeks.

### 3.2. Radiography

**Figure 3:**
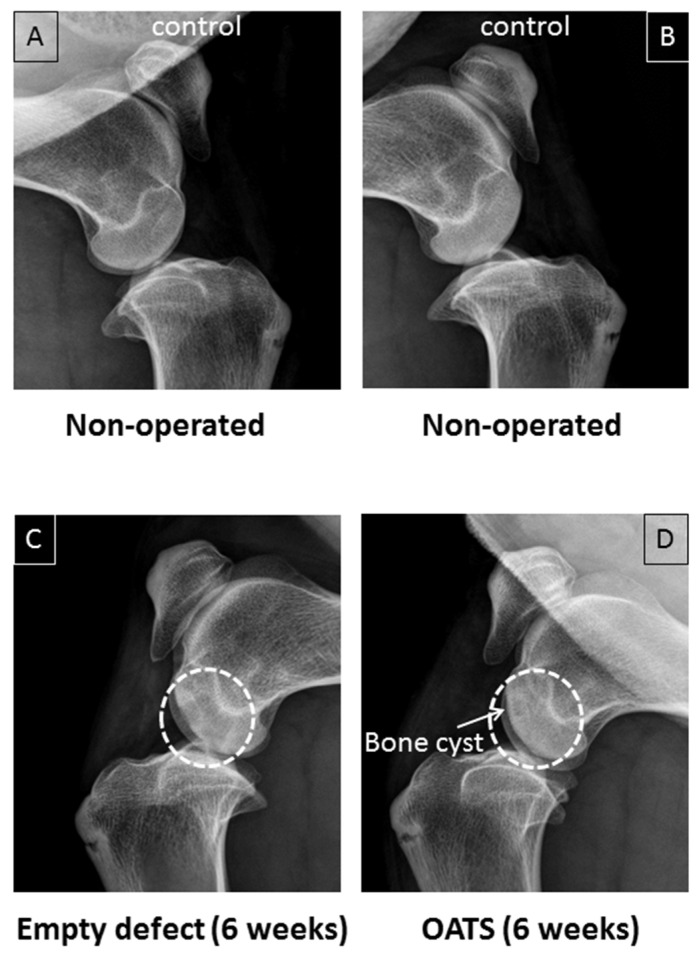
Conventional X-ray. Paired non-operated control stifle joints (**A**,**B**) in comparison to operated joints with 2 empty defects (**C**) or 2 defects refilled with the extracted OATS cylinder (**D**) from the same animal, respectively; white dashed circles in (**C**,**D**) indicate the original defects, the white arrow in (**D)** indicates a bone cyst; all images are 6 weeks after surgery.

Non-operated stifle joints showed normal bone density (*n* = 2 joints from two animals; Figure 3A,B). Six weeks after surgery, in contrast, one operated joint still showed two empty defects as cylinders of reduced bone density (Figure 3C). Defects refilled with OATS cylinders, on the other hand, resembled non-operated joints, except for occasional bone cysts (*n* = 1 joint with 2 defects; Figure 3D).

### 3.3. Gross Morphological Observation and Micro-CT

**Figure 4:**
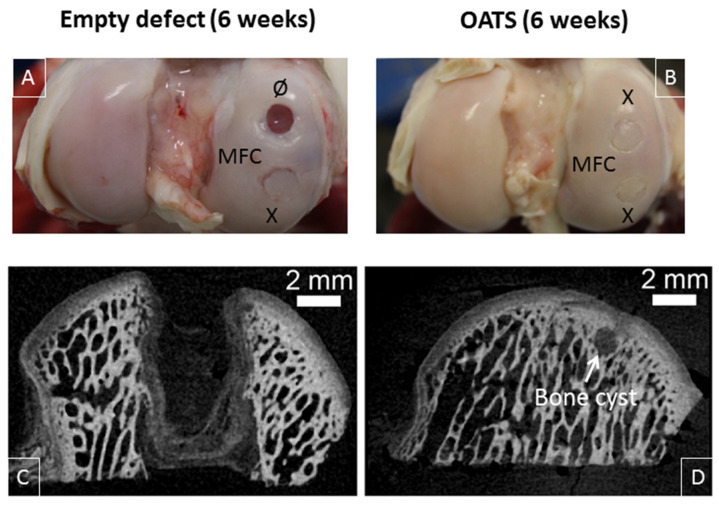
Visual inspection and micro-microcomputed tomography (micro-CT). Representative medial femoral condyles (MFC) containing either one empty defect and one defect refilled with the extracted OATS-cylinder (**A**) or two defects refilled with the extracted OATS cylinder (**B**); micro-CT images of an empty defect (**C**) or a defect refilled with the extracted OATS cylinder (**D**); X indicates original OATS-filled defects; Ø the empty defects; the white arrow a bone cyst; all images 6 weeks after surgery.

Six weeks after surgery, empty defects were still clearly visible (two empty defects; *n* = 2 joints from two animals; Figure 4A). In contrast, the bone part of the defects refilled with original OATS cylinders was already well integrated into the surrounding bone, but the original edges of the cartilage punch were still discernible (six OATS defects; *n* = 4 joints from two animals; Figure 4A,B).

This was confirmed by micro-CT, showing that low radio-density tissue filled most of the empty defect (Figure 4C), whereas the bone part of the reinserted, radio-dense OATS cylinder filled the OATS defect almost completely; only minor, low-density regions and occasional bone cysts surrounded the edges of the OATS cylinder (Figure 4D).

### 3.4. Histology

#### 3.4.1. Empty Defects

**Figure 5:**
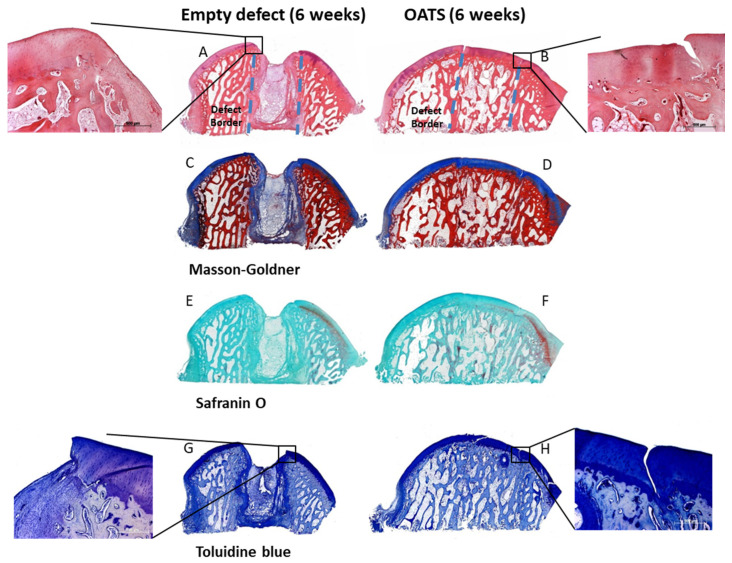
Histology. Hematoxylin/eosin (**A**,**B**), Masson Goldner (**C**,**D**), Safranin-O (**E**,**F**), and Toluidine blue Staining (**G**,**H**) of an empty defect (**A**,**C**,**E**,**G**) or a defect refilled with the extracted OATS cylinder (**B**,**D**,**F**,**H**); original magnification in (**A**–**H**) 40×; original magnification in the insets for (**A**,**B**,**G**,**H**) 100×; original defect borders are indicated by dashed lines in (**A**,**B)**; all stainings 6 weeks after surgery.

Empty defects were histologically discernable, with predominantly disorganized fibrous tissue and some regions of endochondral ossification at the defect edges (Figure 5A,C,E,G). Empty defects showed almost no subchondral bone and cartilage defect healing, but several layers of incompetent connective repair tissue around the defect, which formed a concave surface adjacent to the original articular cartilage (Figure 5A,C,E,G). Whereas Safranin-O staining suggested a substantial reduction in cartilage proteoglycan content at the defect boundaries and the surface of the entire articular cartilage (Figure 5E), Toluidine blue staining only showed limited proteoglycan depletion (Figure 5G). This may be due to the facts that: (i) Toluidine blue has a higher affinity for the sulfur in cartilage compared to Safranin O; and (ii) Safranin O staining may not be a very sensitive indicator of proteoglycan content in cartilage in which glycosaminoglycans have been depleted.

**Figure 6:**
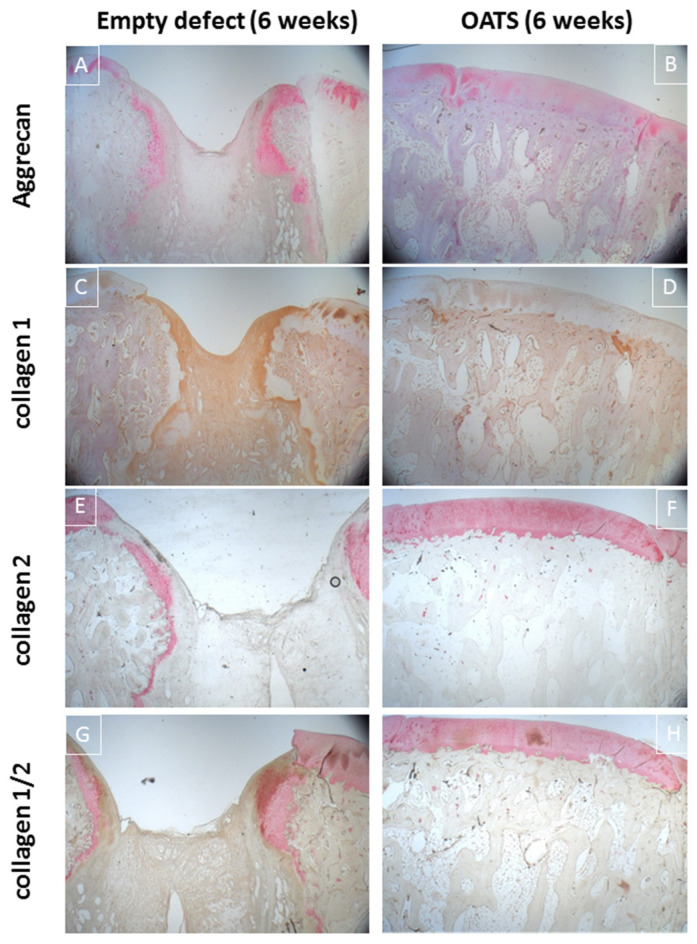
Immunohistology. Single staining for aggrecan (red; **A**,**B**), collagen 1 (brown; **C**,**D**), collagen 2 (red; **E,F**) or double-staining for collagen 1 (brown; **G**,**H**) and collagen 2 (red; **G**,**H**) in an empty defect (**A**,**C**,**E**,**G**) or a defect refilled with the extracted OATS cylinder (**B**,**D**,**F**,**H**); original magnification 12.5×; all stains 6 weeks after surgery.

Immunohistology confirmed incomplete healing of the empty defect, with signs of collagen 1 expression in connective repair tissue (Figure 6C) and heterotopic aggrecan/collagen 2 expression at the edge of enchondral ossifications (Figure 6A,E,G). Double-staining for collagen I/collagen II showed largely separate staining, except for double-stained regions at the inner edge of enchondral ossifications (Figure 6G).

#### 3.4.2. Refilled Defects

The intensely stained hyaline surface of the OATS plugs was smooth and intact. However, there was virtually no chondral integration of the OATS plugs into the adjacent native articular cartilage, resulting in substantial clefts and cartilage degeneration (Figure 5B,D,F,H). In contrast, the subchondral bone component of the OATS cylinder had already integrated almost completely into the original defects (Figure 5B,D,F,H). As in the case of empty defects, Safranin-O staining indicated a substantial proteoglycan reduction in the articular cartilage with reinserted OATS cylinders (Figure 5F), while Toluidine blue staining only showed limited proteoglycan depletion (Figure 5G).

Immunohistology confirmed homogeneous, orthotopic, and distinct staining for collagen 2 and, to some degree, for aggrecan in the articular cartilage. Staining for collagen 1 was restricted to the trabeculae of the subchondral bone (Figure 6B,D,F,H). Double-immunohistology showed completely separate staining for collagens 1 and 2 (Figure 6H).

## 4. Discussion

### 4.1. The Minimally-Invasive, Large Animal OC Defect Model

A minimally-invasive, large animal OC defect model was established in sheep. The present study is the first systematic peri-operative characterization of the model, showing short surgery duration, low wound score, absence of infections or patella luxations, and very rapid postoperative recovery. This model shows both common features and differences with previous models [9,13,14,15,16,18,19,20,33,34,35,36], as summarized in Table 1. Comparison with a classic, not minimally-invasive, medial parapatellar approach was impossible, since serious postoperative complications in this approach ([20] and references therein) prevented permission of the animal study.

### 4.2. Comparison with Published Models

Common features with previous sheep or goat models are: (i) The adult stifle joint as a model for the human knee joint [9,11,12]; (ii) the supine position [18,19]; (iii) a freely movable leg ([20]; present study); and (iv) empty critical-size-defects as controls [18,19,37,38,39], which are also suitable as follow-up locations for donor-site morbidity following OATS [23,25,37]. However, major issues with the choice of a model are unilateral or bilateral arthrotomy and the number of MFC defects (Table 1).

The present unilateral approach may be preferable since it allows immobilization of the operated leg and simulation of partial weight-bearing for three to five weeks in humans. This approach also permits cast application to prevent early implant dislodging and provides one pain-free hind leg to facilitate postoperative rising, standing, and mobilization [20]. In addition, protection of the skin incision and early mobilization reduces the risk of postoperative infections in contaminated sheep pens. Another advantage is the ease of functional assessments based on side-to-side comparisons.

Current use of two adjacent OC implant sites on the main weight-bearing region of the MFC may provide highly comparable biomechanical conditions and may reduce the number of required animals, although defects may break at the cartilage or bone level [37]. However, a significantly faster recovery in the OATS group versus the empty defect group indicated sufficient defect stabilization by an established treatment protocol for OC defects.

A standardized OC punch was used to create the two critical-size-defects. This may be preferable to drilling since it results in cleaner cuts, more distinct margins, and a flatter defect base, improving the conditions for (press-fit) implantation of OATS cylinders or biphasic OC implants [40]. In addition, an OC punch eliminates the need for cooling of the drill to prevent thermal necrosis and/or damage to adjacent cartilage and bone tissue [40].

The tissue-sparing mini-arthrotomy (skin cut 3–4 cm; deep incision 2–3 cm) provided sufficient access to the weight-bearing part of the MFC (Table 1; [18,19,20]). In addition, quadriceps muscle and medial patellar retinaculum were spared to avoid patella instability and/or luxation. Finally, intraoperative patella luxation was not required since a freely moving leg allowed a “mobile window” for full MFC exposure [20]. This is particularly important since goats and sheep show an anatomical predisposition for patellar luxation [41].

An established impairment score was used [31,32] since classic clinical scores are usually not applicable in sheep. However, the present score compared well with a visual analog system [32] and used gradings also applied in the International Knee Documentation Committee (IKDC) score (e.g., normal, nearly normal, abnormal, or severely abnormal [26,42]).

Focal experimental cartilage or OC defects for the evaluation of new implants are preferably created on the MFC, lateral femoral condyle (LFC), or trochlea of the stifle joint. Based on high cartilage thickness and easy surgical access, the center of the MFC in sheep appears to be the most attractive site to place defects ([43]; and references therein). Therefore, the majority of the in vivo studies in sheep have used the MFC, either alone or combined with other implant sites ([43]).

In contrast, the cartilage thickness on the LFC is consistently lower than on the MFC, theoretically making it a less attractive defect site [43]. Indeed, it has been used less frequently than the MFC for cartilage repair studies, possibly also because the tendon of the musculus extensor digitorum longus covers the whole LFC in a ventral-dorsal direction ([43]; and references therein).

The cartilage thickness on the trochlea is also considerably lower than that on the MFC and LFC. However, the femoral trochlea has been frequently applied for cartilage defect studies in sheep, goat, and mini-pig [43], possibly due to its large surface area, which allows the generation of multiple chondral or osteochondral defects or different implant fixation techniques [43].

### 4.3. Comparison with the Sham Group

Empty defect and OATS animals reached a postoperative impairment score of 0 significantly later than sham animals (6 and 2.5 days, respectively), likely due to more extended joint pain/discomfort. Thus, the inclusion of a sham group in the present study provides novel evidence for specific aspects of the functional recovery, i.e., the relative contribution of operation/anesthesia and defect generation.

In addition to its function as a critical-size-defect, the empty defect may also serve as a follow-up location for donor-site morbidity following OATS [21,22,23,24,25]. Although incomplete filling of empty defects with connective scar tissue has been reported previously on the basis of visual inspection or X-ray/MRI in animal and human studies ([22,25]; present study), detailed structural and molecular analysis of the temporal developments at the edge of the empty/donor site is only possible in experimental animal studies ([25,37]; present study). Thus, the present large animal model for OC defects is also suitable for the detailed analysis of donor site morbidity. In fact, the empty defect showed incomplete healing with dedifferentiation or heterotopic differentiation after six weeks, as well as the occasional occurrence of subchondral cavitary lesions, in good agreement with previous reports on this topic [25,37].

## Figures and Tables

**Table 1:** Comparison of large animal OC defect models. Abbreviations: ß-TCP = ß-Tricalcium phosphate; OATS = osteochondral autologous transplantation system; n.a. = not available; wound score: 0 = no signs of wound secretion, swelling or infection; 1 = signs of wound secretion; 2 = signs of wound swelling/redness; 3 = signs of swelling/redness and wound secretion; 4 = signs of wound secretion, swelling/redness, and infection.

	Mayr HO [18]	Bernstein A [19]	Orth P [20]	Present Study
**Sheep** **(gender; age)**	Merino (female; 2–4 years)	Merino (female; 2–4 years)	Merino (female; 2–4 years)	Merino (female; 2–6 years)
**Position**	Supine; leaning _˜_30° to caudal	Supine; leaning _˜_30° to caudal	Supine position	Supine position
**Invasiveness**	Mini-arthrotomy (3 cm cut)	Mini-arthrotomy (3 cm cut)	Mini-arthrotomy (4–5 cm cut)	Mini-arthrotomy (3–4 cm skin cut; 2–3 cm deep incision)
**Stifle joint**	Bilateral	Bilateral	Bilateral	Right side
**Defect location**	Medial femoral condyle (1 defect; center of load-bearing area)	Medial femoral condyle (1 defect; center of load-bearing area)	None; (exposure; lateral + medial trochlear facet; medial + lateral femoral condyle; menisci)	Medial femoral condyle (2 defects; anterior + central part of load-bearing area)
**Critical-size** **OC defect(s)**	Diameter: 7 mm; Depth: 25 mm	Diameter: 7 mm; Depth: 25 mm	n.a.	Diameter: 7 mm; Depth: 10 mm
**Specific tools**	Drill (with fluid cooling)	Drill (with fluid cooling)	-	OATS punch, (Arthrex, Munich, Germany)
**Implant** **(size; modification)**	ß-TCP implant (diameter:7 mm; depth: 25 mm; seeded with autologous chondrocytes); empty control	ß-TCP implant (diameter: 7 mm; depth: 25 mm; seeded with autologous chondrocytes); empty control	none	OATS cylinder (diameter: 6 mm; depth: 10 mm); empty control
**Operation/** **anesthesia** **duration**	Not determined	Not determined	Approximately 20 min/ Not determined (no defect/implant)	Approximately 0.5 h/ approximately 1.4 h
**Wound score;** **days 7 and 14**	Not determined (2 superficial; 1 deep infection)	Not determined	Not determined (no deep wound infections or empyema)	0.4 and 0.1 (of max. 4)
**Time to complete stand**	Not determined (normal walking at 42 d)	Not determined	1–2 h	1.5 h
**Immobilization**	None (full weight-bearing + range of motion)	None (full weight-bearing + range of motion)	None (full weight-bearing + range of motion)	1 week; (fiber glass cast)
**Follow-up time** **(weeks)**	6, 12, 26, 52	6, 12, 26, 52	26 (6 months)	6

## Abbreviations

ALP	alkaline phosphatase
BSA	bovine serum albumin
β-TCP	β-Tricalcium phosphate
DAB	diaminobenzidine
HE	hematoxylin and eosin
HRP	horseradish peroxidase
Ig	immunoglobulin
IKDC	International Knee Documentation Commitee
MFC	medial femoral condyle
LFC	lateral femoral condyle
OATS	osteochondral autologous transplantation cylinder
OC	osteochondral
TBS	tris-buffered saline
